# CTLA-4 haploinsufficiency presenting with chronic myeloid leukemia, bullous pemphigoid, and PLA2R-positive membranous nephropathy: a case report

**DOI:** 10.1186/s13223-026-01011-7

**Published:** 2026-01-25

**Authors:** Nouraldeen Deeb, Salahaldeen Deeb, Fares Sayed Ahmed, Younis Malik Younis Amro, Abdallah Altell, Aliaa khalili

**Affiliations:** Faculty of Medicine, Al-Quds University, East Jerusalem, Occupied Palestinian Territory

**Keywords:** Case report, CTLA-4 haploinsufficiency, Chronic myeloid leukemia (CML), Membranous glomerulonephritis (MGN), Paraneoplastic syndromes

## Abstract

**Background:**

Cytotoxic T-lymphocyte–associated protein 4 (CTLA-4) haploinsufficiency is a primary immune-regulatory disorder characterized by T-cell overactivation and multisystem autoimmunity. Malignancies, particularly lymphomas and gastric cancer, have been reported in approximately 12–13% of individuals with CTLA-4 haploinsufficiency, but associations with chronic myeloid leukemia (CML) are very rarely described. We report a young adult with genetically and functionally confirmed CTLA-4 haploinsufficiency who developed a triad of BCR-ABL1–positive chronic myeloid leukemia (CML), bullous pemphigoid, and PLA2R-positive membranous glomerulonephritis (MGN), highlighting diagnostic and management lessons across immunology, hematology, dermatology, and nephrology.

**Case presentation:**

A 21-year-old woman with Hashimoto thyroiditis and iron-deficiency anemia was found to have BCR-ABL1–positive CML. Within months she developed recurrent infections and paraneoplastic blistering disease. Whole-exome sequencing detected a heterozygous CTLA4 c.529_530insT (p.Tyr177LeufsTer2) frameshift; flow cytometry showed reduced CTLA-4 expression, establishing functional haploinsufficiency. Six months later she presented with edema and nephrotic-range proteinuria; serology revealed markedly elevated anti-PLA2R antibodies, and kidney biopsy confirmed immune-complex MGN. Management included a TKI (imatinib/nilotinib as indicated over the course), low-dose corticosteroids, monthly IVIG for infection/immune modulation, and rituximab for bullous disease and MGN. This resulted in molecular remission of CML, resolution of skin lesions, and sustained normalization of proteinuria over 12 months.

**Conclusions:**

The convergence of CML, bullous pemphigoid, and PLA2R-positive MGN in CTLA-4 haploinsufficiency broadens the clinical phenotype and underscores the importance of considering inborn errors of immunity in young adults with refractory, multisystem autoimmunity and hematologic abnormalities. Early genetic diagnosis can guide targeted immunomodulation and organ preservation, and multidisciplinary care is essential for optimal outcomes.

**Supplementary Information:**

The online version contains supplementary material available at 10.1186/s13223-026-01011-7.

## Introduction

Cytotoxic T-lymphocyte–associated protein 4 (CTLA-4) is an inhibitory immune checkpoint receptor that downregulates T-cell activation. Germline heterozygous loss-of-function variants in CTLA4 cause CTLA-4 haploinsufficiency, a primary immune-regulatory disorder that can present with cytopenias, inflammatory bowel disease, and organ-specific autoimmune disorders such as type 1 diabetes and autoimmune thyroiditis [1, 2]. Affected individuals typically develop autoimmune disease accompanied by polyclonal T-cell proliferation, highlighting the central role of CTLA-4 in maintaining peripheral tolerance. CTLA-4 function has also been targeted therapeutically using CTLA-4 immunoglobulin (Ig) fusion proteins [3]. 

Chronic myeloid leukemia (CML) is a type of myeloproliferative neoplasm caused by a reciprocal translocation between chromosomes 9 and 22, known as the Philadelphia chromosome, which results in the formation of BCR/ABL1 fusion gene. This fusion gene encodes a hybrid tyrosine kinase with increased enzymatic activity, leading to uncontrolled proliferation of hematopoietic stem cells [4]. CML progresses through three phases: chronic, accelerated, and blast phases. The advent of BCR/ABL1 tyrosine kinase inhibitors (TKIs) has significantly improved overall survival rates for individuals in the chronic phase of CML [5].

Membranous glomerulonephritis (MGN) is one of the most common causes of nephrotic syndrome, is a relatively rare disease. It is characterized by basement membrane thickening and subepithelial immune deposits without cellular proliferation or infiltration. MGN can be classified as either primary (idiopathic) or secondary, with the latter being associated with underlying conditions such as autoimmune diseases (e.g., systemic lupus erythematosus, rheumatoid arthritis), infections, malignancies, or drug-induced reactions [6]. In addition, immune-regulatory disorders such as CTLA-4 haploinsufficiency may contribute to a predisposition to glomerular autoimmunity [6].

## Case report

A 21-year-old woman with a medical history of Hashimoto’s thyroiditis and iron deficiency anemia presented to the emergency department on October 23, 2024, with complaints of persistent vomiting and a progressively spreading skin rash. The rash, which began two days before her admission, initially affected her hands, but later spread extensively. Despite its widespread nature, the rash was neither pruritic nor painful, The chronological sequence of key clinical events, investigations, treatments, and outcomes is summarized in (Table 1).

**Table 1:** Timeline, of the episode of care

Date/period	Event
~Aug–Oct 2024	Two-month history of cough, dyspnea, intermittent fevers.
2024-10-21 (patient-reported)	Rash onset 2 days prior to admission.
10/23/2024	ED presentation with vomiting and rapidly spreading rash; labs consistent with leukocytosis, AKI and TLS; CML subsequently diagnosed (BCR-ABL1 70%).
During hospitalization	Blistering disease confirmed on skin biopsy (paraneoplastic pemphigus); MRSA skin infections; severe Pseudomonas pneumonia → ARDS treated with amikacin.
Genetics	WES identifies heterozygous CTLA4 c.529_530insT (p.Tyr177LeufsTer2).
Imaging	PET-CT: avid axillary nodes; splenomegaly; benign axillary node histology.
~ 6 months post-CML dx (Renal)	Facial edema; +3 proteinuria; albumin 1.6 g/dL; anti-PLA2R 360 RU/mL → biopsy-proven MGN.
Therapy	Nilotinib; IVIG monthly; hemodialysis ×3 for TLS; rituximab for skin disease; low-dose steroids + cyclosporine for MGN.
Follow-up	Clinical improvement in pulmonary and cutaneous disease; ongoing monitoring planned.

Key presentations, diagnostics, treatments, and outcomes by date

ED, emergency department; AKI, acute kidney injury; TLS, tumor lysis syndrome; WES, whole-exome sequencing; PET-CT, positron emission tomography–computed tomography; MGN, membranous glomerulonephritis; IVIG, intravenous immunoglobulin; TKI, tyrosine kinase inhibitor; ARDS, acute respiratory distress syndrome; MRSA, methicillin-resistant Staphylococcus aureus

Upon hospital admission, routine laboratory investigations were performed. which include CBC, that showed leukocytosis, acute renal failure suggested by a very high Creatinine, and findings of tumor lysis syndrome (TLS) with extremely high uric acid, as shown in Table 2, which resolved after three sessions of hemodialysis, she was continued on allopurinol after stabilization a diagnosis of chronic myeloid leukemia (CML) was subsequently made. Initially, a peripheral blood smear showed monocytosis with a left shift, raising concerns of acute myeloid leukemia (AML), However, bone marrow biopsy findings demonstrated hypercellular marrow with 7% blasts, PCR testing confirmed the presence of the BCR-ABL1 fusion gene (70%), establishing the diagnosis of CML. This presentation was further complicated by TLS, as summarized in Table 2.

**Table 2:** Laboratory results: initial labs before therapy show marked leukocytosis with anemia and thrombocytopenia; severe hyperuricemia, acute kidney injury, and metabolic acidosis; hyperphosphatemia, hyperkalemia, and hypocalcemia—findings consistent with tumor Lysis syndrome

Test	Result	Normal range	Abnormality
WBC	105,000/µL	4000–11,000/µL	Elevated
Granulocytes	85%	50–70%	Elevated
Hemoglobin	9.0 g/dL	12–15 g/dL	Decreased
Platelets	57,000/µL	150,000–450,000/µL	Decreased
Uric acid	37 mg/dL	3.4–7.0 mg/dL	Elevated
Creatinine	7.0 mg/dL	0.7–1.3 mg/dL	Elevated
ABG (pH)	7.2	7.35–7.45	Decreased
IgE level	153.4 IU/mL	< 100 IU/mL	Elevated
Eosinophils	11.60%	1–6%	Elevated
PO_4_	18 mg/dl	2.8–4.5 mg/dl	Elevated
K	5.6 mmol/L	3.6–5.2 mmol/L	Elevated
Ca	6.5 mg/dl	8.6–10.3 mg/dl	Decreased

Elevated IgE and eosinophilia were also noted

WBC, white blood cell count; IgE, immunoglobulin E; ABG, arterial blood gas; PO_4_, phosphate; K, potassium; Ca, calcium; AKI, acute kidney injury; TLS, tumor lysis syndrome

Prior to this admission, The patient reported recurrent skin infections with methicillin-resistant Staphylococcus aureus (MRSA) in the past, confirmed by swab culture, characterized by erythematous lesions, ulcerations, and bullae. Additionally, over the preceding two months, she had experienced persistent cough, shortness of breath, and intermittent fevers.

**Fig. 1:**
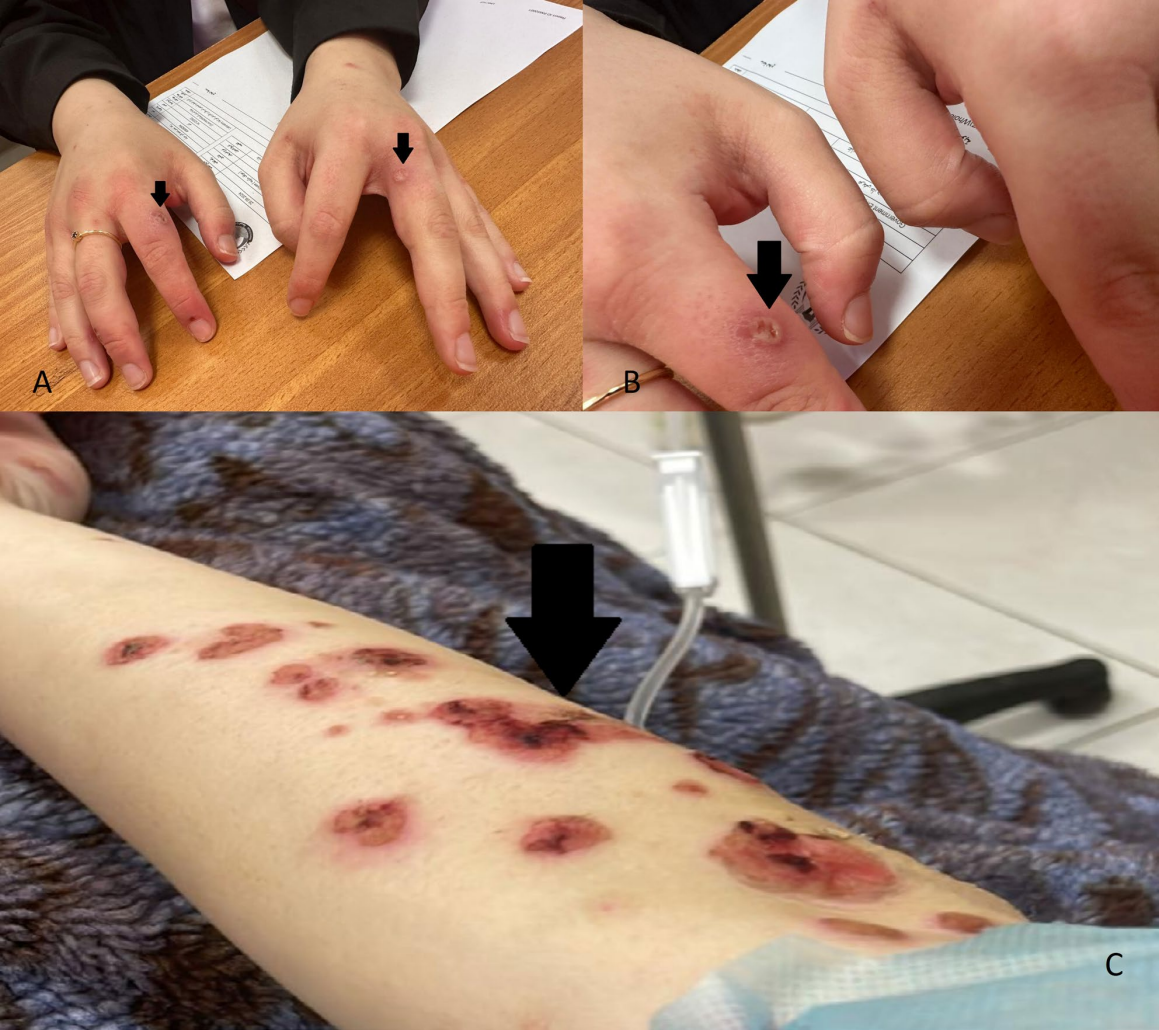
**A** and** B** The hands of the patient, arrows shows pemphigus and erythematous lesions,** C** The arm of the patient, arrow shows pemphigus and erythematous lesions after spreading

During her index hospitalization for CML and tumor lysis syndrome, physical examination revealed hyperemic lesions and bullae over her extremities and torso, along with erosions in her oral cavity suggestive of paraneoplastic pemphigus as shown in (Fig. 1). A punch biopsy of a skin lesion confirmed the diagnosis, demonstrating focal spongiosis and an intraepidermal suprabasal blister filled with fluid and mixed inflammatory cells. Splenomegaly measuring 15.5 cm was palpable, cervical and axillary lymphadenopathy were observed, and a 1.5 × 1.5 cm mass was identified in the lower outer quadrant of the left breast. Respiratory examination revealed bilateral crackles.

During the same hospitalization, Her clinical course was complicated by a severe episode of pneumonia caused by multidrug-resistant Pseudomonas aeruginosa leading to moderate acute respiratory distress syndrome (ARDS) Which required hospitalization and treatment with amikacin. She also had a history of oral candidiasis, two episodes of otitis media, and recurrent sinusitis, all of which further complicated her clinical picture.

The patient also had recurrent herpes oralis, treated with acyclovir due to a documented allergy to valacyclovir. This presentation posed several challenges. Markedly elevated IgE and eosinophilia with recurrent infections initially raised concern for hyper-IgE syndrome, potentially obscuring an underlying immune-regulatory defect; targeted evaluation ultimately favored CTLA-4 haploinsufficiency after genetic testing. The coexistence of BCR-ABL1–positive CML and a blistering disorder (biopsy-proven paraneoplastic pemphigus) complicated attribution of symptoms to malignancy versus primary immune dysregulation. In addition, extensive lymphadenopathy with benign histology on excision and PET-avid nodes required correlation across imaging, histopathology, and serology. Finally, anti-PLA2R–positive nephrotic-range proteinuria and biopsy-confirmed membranous glomerulonephritis introduced a parallel autoimmune renal process requiring careful differentiation from malignancy-associated nephropathy and treatment-related effects., as shown in Table 2.

However, whole-exome sequencing (WES) revealed a heterozygous duplication, c.529_530insT, in exon 3 of CTLA4 (chr2: 203 871 442), producing a frameshift and premature stop codon (p.Tyr177LeufsTer2). The variant was detected with solid technical support (allele frequency ≈ 38%, 41/107 reads) and is currently listed in ClinVar as a variant of uncertain significance (VUS). This mutation is associated with immune dysregulation, leading to autoimmune manifestations, lymphoproliferative disorders, and increased susceptibility to recurrent infections. The patient denied smoking or alcohol consumption.There was no family history of hematologic malignancies or immunodeficiencies.

**Fig. 2:**
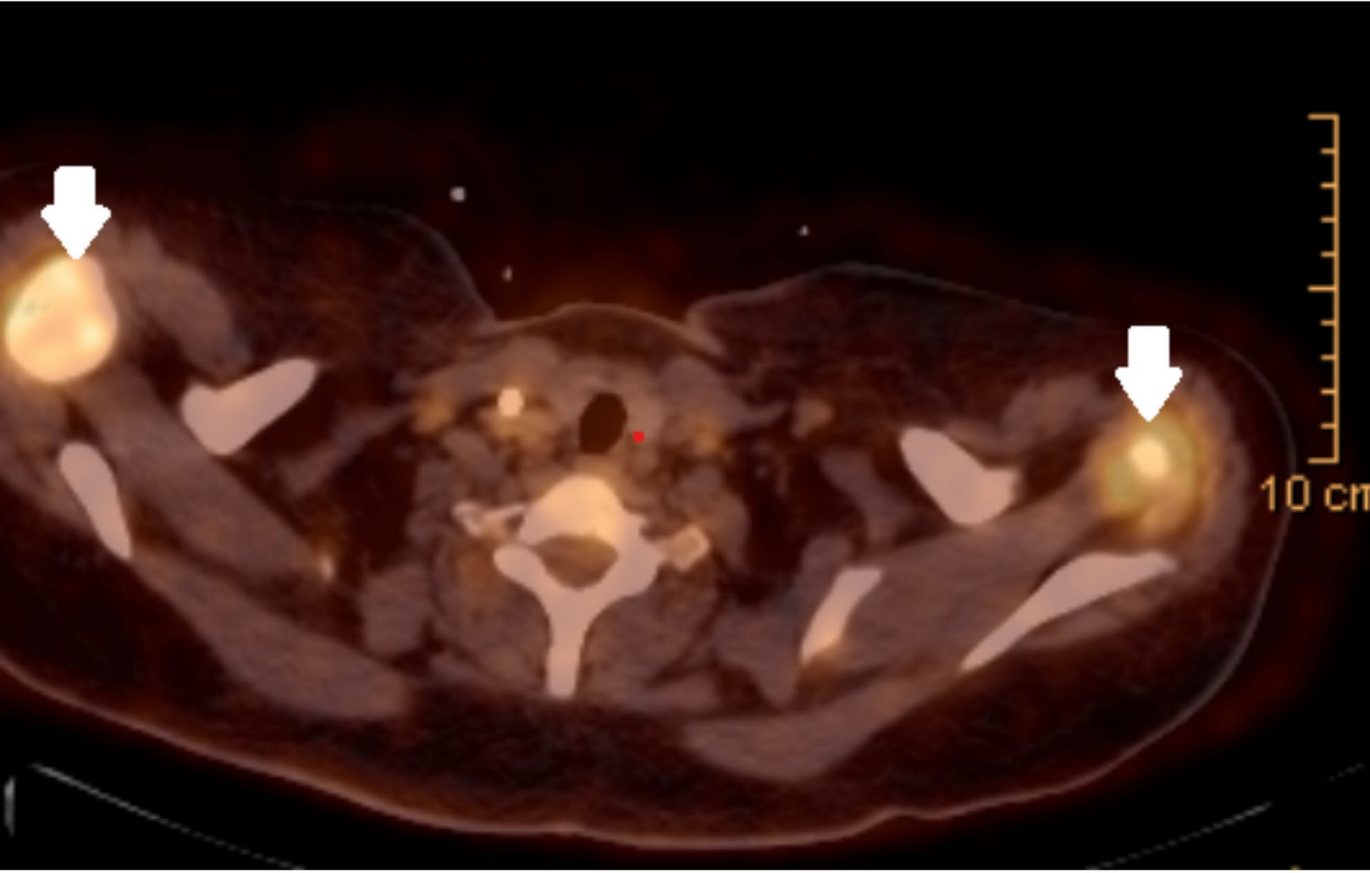
PET-CT showing enlarged axillary lymph nodes with increased FDE uptake

Imaging studies, including whole-body CT and PET-CT, demonstrated significant lymphadenopathy in cervical, axillary, and retroperitoneal regions, along with splenomegaly and a breast mass, with PET-CT revealing intense FDG uptake in the axillary lymph nodes (SUV 7.9, Fig. 2), and spleen (SUV 6.5),with no osseous deposits were observed. A left axillary lymph node excision was performed, and histopathological examination revealed non-Malignant reactive lymph nodes with follicular hyperplasia and increased Eosinophilic infiltration. The patient also had a history of acute kidney injury (AKI) and recurrent urinary tract infections (UTIs) with Klebsiella species. Approximately six months after the CML diagnosis, she developed early morning facial puffiness and lower limb edema. Urinalysis showed + 3 proteinuria and + 1 red blood cells ( RBC), findings that were refractory to bolus corticosteroid therapy. Her serum albumin was markedly low at 1.6 g/dL.

Further evaluation demonstrated a significantly elevated anti-PLA2R antibody: (360 RU/ml, normal range is < 20 RU/ml), indicating primary autoimmune membranous Glomerulonephritis (MGN). The diagnosis was confirmed by kidney biopsy adding further complexity to her already multifaceted clinical picture.

## Management and multidisciplinary approach

The patient was initiated on Nilotinib (Tasigna) for the management her chronic myeloid leukemia (CML). Monthly high-dose intravenous immunoglobulin (IVIG) therapy was used to address her recurrent infections and modulate immune dysregulation. Her Multidrug-resistant Pseudomonas pneumonia was successfully treated with a 10-day course of amikacin.

For her paraneoplastic pemphigus and her associated dermatologic lesions, she received Rituximab therapy, which led to notable improvement in her skin lesions. Tumor lysis Syndrome(TLS) was managed with three hemodialysis sessions followed by ongoing hydration and allopurinol therapy, Additionally she is on low dose steroids and cyclosporin for treatment of membranous glomerulonephritis.

At follow-up visit, the patient demonstrated significant clinical improvement. Her pulmonary symptoms related to Pseudomonas had resolved without recurrence. The skin lesions associated with paraneoplastic pemphigus also showed marked resolution following rituximab therapy. Despite a favorable overall clinical response and good adherence to treatment, without any reported issues related to tolerability or treatment discontinuation. her underlying CTLA-4 haploinsufficiency and newly diagnosed primary membranous glomerulonephritis remain ongoing concern, these conditions require ongoing monitoring and targeted immunotherapy to prevent future complications and recurrent infections.

Prognosis. Short-term outlook is favorable given molecular remission of CML on TKI therapy, resolution of blistering disease after rituximab, and sustained normalization of proteinuria at 12 months. Long-term risk remains shaped by CTLA-4 haploinsufficiency, which confers susceptibility to infections, autoimmune flares, and certain malignancies; continued surveillance and immunomodulation are warranted.

## Discussion

To the best of our knowledge, this is the first reported case of a unique clinical presentation combining chronic myeloid leukemia (CML) with an underlying primary immunodeficiency linked to Cytotoxic T-lymphocyte-associated protein 4 (CTLA-4) haploinsufficiency, further complicated by membranous glomerulonephritis (MGN) and bullous pemphigoid. The patient, a 21-year-old female, initially presented with features consistent with CML and was subsequently diagnosed following standard hematological and molecular assessments. Further investigations, prompted by persistent immune dysregulation and atypical clinical findings, led to the identification of CTLA-4 haploinsufficienc. Additionally, the patient developed nephrotic-range proteinuria, and renal biopsy confirmed the presence of MGN, suggesting a possible immunological interplay between these conditions. This rare and complex combination underscores significant diagnostic and therapeutic challenges, requiring a multidisciplinary approach for optimal management. In this report, we aim to delineate the diagnostic process, therapeutic strategy, and clinical course, contributing to the growing understanding of the intersection between hematological malignancies, primary immunodeficiencies, and glomerular diseases.

The case study presents a rare and complex clinical scenario involving a patient with a CTLA-4 mutation and concurrent Chronic Myeloid Leukemia (CML) who developed bullous pemphigoid (BP) alongside iron deficiency anemia, recurrent infections, Hashimoto thyroiditis, and primary glomerulonephritis. Each of these conditions is significant on its own, however, their coexistence highlights a complicated interplay between Hematologic malignancy, autoimmunity and immune dysregulation. This unique constellation of findings necessitates a comprehensive diagnostic workup and multidisciplinary management approach.

CTLA-4 is an immune checkpoint receptor that plays a crucial role in immune homeostasis by downregulating T-cell activation. Germline mutations in the CTLA4 gene can cause CTLA-4 haploinsufficiency through loss-of-function or dominant-negative effects, resulting in the clinical syndrome historically referred to as ‘CTLA-4 insufficiency’ [7]. This disorder is characterized by a wide spectrum of clinical manifestations, including autoimmune diseases (e.g., systemic lupus erythematosus, type 1 diabetes, autoimmune enteropathy) and immunodeficiency with recurrent infections. The clinical presentation of CTLA4 mutations varies according to genotype; loss-of-function variants impair the receptor’s ability to downregulate immune activation, leading to hyperactive immune responses and a predisposition to autoimmune disease [7, 8].

Although CTLA-4 mutations are rare, although they have been implicated to numerous autoimmune disorders. These include Type 1 diabetes, autoimmune thyroiditis, rheumatoid arthritis, inflammatory bowel disease, Systemic Lupus Erythematosus (SLE), and Alopecia Areata (AA) which is an autoimmune condition characterized by non-scarring hair loss due to T-cell–mediated destruction of hair follicle [1]. While some autoimmune disorders may result from de novo mutations, CTLA-4 mutations are mainly inherited in an autosomal dominant fashion, Heterozygous loss-of-function mutations account for the majority of cases, whereas homozygous or compound heterozygous forms may result in more severe or early onset phenotypes [9].

Research on CTLA- 4 gene variants (notably + 49 A/G and − 318 C/T) shows that certain alleles are enriched in individuals with autoimmune diseases. The + 49G allele in rs231775 has been associated with increased risk of RA, SLE, and type 1 diabetes in Caucasian and Asian populations [10].

A study involving 184 CTLA-4 mutation carriers revealed that 131 had exhibiting symptoms, Among the symptomatic individuals, 17 cases of malignancy were reported, representing a cancer prevalence of 12.9%. The types of malignancies included 10 lymphomas, five gastric cancers, one multiple myeloma, and one metastatic melanoma. Notably, Epstein-Barr Virus (EBV) was associated with a significant proportion of these malignancies—specifically, 7 out of 10 lymphomas and 3 out of 5 gastric cancers [11].

Although CTLA-4 mutations are rare, they are associated with an increased risk of malignancy, particularly those linked to EBV. However, there are currently no peer-reviewed studies establishing a connection between CTLA- 4 mutations to CML. The condition’s incomplete penetrance and variable expressivity highlight the need for more research to better understand its epidemiology and pathophysiology.

Whole-exome sequencing of the patient revealed a CTLA-4 mutation located on chromosome 2, at cytogenetic location 2q33.2 with genomic coordinates (GRCh38) between 2:203,867,771 − 203,873,965. This specific mutation is associated with several autoimmune and immune dysregulation disorders, including Celiac disease, DM type1, SLE, Hashimoto thyroiditis, and other forms of immune dysregulation [12].

Chronic myeloid leukemia (CML) is a myeloproliferative neoplasm arising from a clonal expansion of hematopoietic stem cells [13]. It is characterized by uncontrolled proliferation of myeloid lineage cells and is most commonly driven by the BCR-ABL1 fusion gene. This genetic abnormality results from a reciprocal translocation between chromosomes 9 and 22, forming the Philadelphia chromosome (t(9;22)(q34;q11)). The resulting BCR-ABL1 protein exhibits constitutive tyrosine kinase activity, which promotes aberrant cell growth and resistance to apoptosis, forming the molecular basis of CML pathogenesis.

While CTLA-4 mutations are implicated in immune dysregulation syndromes and a range of autoimmune diseases, they have no known association with the development of CML. These two genetic alterations appear to act through independent biological mechanisms—CTLA-4 affecting immune regulation, and BCR-ABL1 driving malignant transformation of hematopoietic progenitor cells.

The incidence of CML varies geographically but is generally estimated at 1–2 cases per 100,000 individuals per year in Western populations. Owing to the efficacy of tyrosine kinase inhibitors (TKIs), survival rates have dramatically improved, leading to a growing prevalence of CML, particularly in developed countries [14].

CML predominantly affects adults between the ages of 50 and 60 and is slightly more common in males than females. Though the disease can occur at any age, pediatric cases remain rare.

Bullous pemphigoid is an autoimmune blistering condition caused by autoantibodies that target dermal-epidermal junction components. The development of BP in a CTLA-4 mutant patient may be due to a susceptibility to autoimmune disorders caused by poor immune regulation [15]. Notably, this literature suggests that people with CTLA-4 mutations are more susceptible to autoimmune conditions, including skin disorders such as BP.

The coexistence of BP and CML adds complexity to the clinical picture, as both the malignancy and its treatments (particularly tyrosine kinase inhibitors) can influence immune function. However, the absence of definitive association between CTLA-4 mutations and CML gives support to the theory that the autoimmune manifestations seen in this patient are more likely attributable to the underlying CTLA-4 mutation than the presence of CML [1].

Recent investigations revealed that our patient is also experiencing nephrotic syndrome, characterized by significant proteinuria, hypoalbuminemia, literature supports a link between CTLA-4 dysfunction and glomerular disease. Notably, the CTLA-4 + 49GG genotype—a high-risk polymorphism—has been associated with minimal change disease (MCD), focal segmental glomerulosclerosis (FSGS), and membranous nephropathy in autoimmune-prone individuals [16, 17].

Additionally, the presence of positive anti-PLA2R antibodies in this patient suggests a possible overlap or transition toward membranous nephropathy, further complicating the clinical picture. These findings imply a potential mechanistic involvement of CTLA-4–mediated immune dysregulation in the pathogenesis of autoimmune glomerulopathies [18]. Consequently, the patient requires close monitoring and carefully tailored immunosuppressive therapy, taking into account the broader context of CTLA-4 haploinsufficiency.

Because it poses difficulties for diagnosis and treatment, the coexistence of bullous pemphigoid in this patient is clinically noteworthy. Corticosteroids and other immunosuppressive medications that target autoimmune pathways are commonly used to treat BP. Immunosuppression must be carefully adjusted in the presence of a CTLA-4 mutation to reduce the possibility of worsening infections or impairing immune surveillance in a patient who also has CML. The renal findings further complicate the patient’s case, necessitating close monitoring and careful immunosuppressive management.

Iron deficiency anemia observed in this patient adds another layer of complexity in this patient. Anemia in such cases may be caused by chronic inflammation, nutritional deficiencies, or gastrointestinal pathology, potentially influenced by immune dysregulation or malignancy-associated factors. Addressing and applying the proper management for this patient’s anemia is critical to reducing morbidity and optimizing the overall health status.

The overall scope of this case study is restricted to a single patient; therefore, generalizations should be made with caution. Although CTLA-4 mutations are becoming more widely acknowledged as causes of immunological dysregulation, it is difficult to make firm conclusions since CML and BP rarely coexist. Furthermore, it is still unclear what mechanisms underlie the co-occurrence of autoimmune diseases, hematological malignancies, and CTLA-4 mutations.

Clarifying the molecular processes by which CTLA-4 mutations affect the development of autoimmune diseases and their relationships with hematological malignancies should be the main goal of future study. To determine if individuals with CTLA-4 haploinsufficiency have a higher risk of developing several autoimmune or hematological disorders, more research and case series are required.

## Patient perspective

“Living with what seemed like three different diseases felt like fighting on several fronts at once. Getting the genetic answer was a relief—it helped me understand why my body kept attacking itself. The combination of the targeted leukaemia pill and immunotherapy turned things around; my skin healed, the swelling in my legs disappeared, and I can plan my future again. I’m grateful the team kept searching until the pieces fit.”

## Conclusion

This case broadens the clinical phenotype of CTLA-4 haploinsufficiency by demonstrating the convergence of BCR-ABL1–positive chronic myeloid leukemia, bullous pemphigoid, and PLA2R-positive membranous glomerulonephritis in a single young adult. The coexistence of hematologic malignancy, multisystem autoimmunity, and immune-mediated nephropathy highlights the importance of considering inborn errors of immunity in young patients who present with unusual or overlapping autoimmune and hematologic manifestations. Early recognition of CTLA-4 haploinsufficiency, together with timely genetic testing, targeted immunomodulation, and coordinated multidisciplinary care, appears crucial to achieving disease control and preserving renal function in such patients.

Further reports and systematic studies are needed to clarify the mechanisms linking CTLA-4 haploinsufficiency to malignancy and glomerular autoimmunity and to guide long-term management strategies.

## Supplementary Information

Below is the link to the electronic supplementary material.

Supplementary Material 1.

## Data Availability

The raw data supporting the conclusions of this article will be madeavailable by the authors, without undue reservation report and any accompanying images. A copy of the written consent is available for review by the Editor-in-Chief of this journal.

## Declarations

### Patient Perspective

The patient reported that her illness had significantly impacted her daily life, especially due to the persistent infections and skin lesions. She expressed relief after receiving a definitive diagnosis and reported noticeable improvement in her symptoms after beginning therapy. Despite the complexity of her condition, she felt supported by the medical team and expressed hope for continued improvement with ongoing care.
