# Supplementary material for: CTLA-4 haploinsufficiency presenting with chronic myeloid leukemia, bullous pemphigoid, and PLA2R-positive membranous nephropathy: a case report
Source: Allergy Asthma Clin Immunol. 2026 Jan 25;22:5. doi: 10.1186/s13223-026-01011-7 (PMC12879419; doi:10.1186/s13223-026-01011-7)

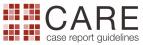

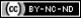

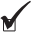
**CARE Checklist of information to include when writing a case report**


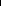


**Topic**

**Title**

**Key Words**

**Abstract**

**(no references)**

**Introduction**

**Patient Information**

**Clinical Findings Timeline**

**Diagnostic Assessment**

**Therapeutic Intervention**

**Follow-up and Outcomes**

**Discussion**

**Patient Perspective Informed Consent**


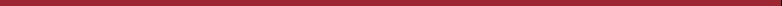


**Item Checklist item description Reported on Line**

**1** The diagnosis or intervention of primary focus followed by the words “case report” . . . . . . . . . . . . . . . . . . . . . . . . . . 1-2

**2** 2 to 5 key words that identify diagnoses or interventions in this case report, including "case report" . . . 15-17 **3a** Introduction: What is unique about this case and what does it add to the scientific literature? . . . . . . . . . . . . . . . . 33-34 **3b** Main symptoms and/or important clinical findings . . . . . . . . . . . . . . . . . . . . . . . . . . . . . . . . . . . . . . . . . . . . . . . . . . . . . . . 29-31, 38-39 **3c** The main diagnoses, therapeutic interventions, and outcomes . . . . . . . . . . . . . . . . . . . . . . . . . . . . . . . . . . . . . . . . . . . 31 ,35-42 **3d** Conclusion—What is the main “take-away” lesson(s) from this case? . . . . . . . . . . . . . . . . . . . . . . . . . . . . . . . . . . . . . 33-37

**4** One or two paragraphs summarizing why this case is unique (**may include references**) . . . . . . . . . . . . 62-77 **5a** De-identified patient specific information. . . . . . . . . . . . . . . . . . . . . . . . . . . . . . . . . . . . . . . . . . . . . . . . . . . . 82-88 **5b** Primary concerns and symptoms of the patient. . . . . . . . . . . . . . . . . . . . . . . . . . . . . . . . . . . . . . . . . . . . . . . . . . . . . 83-87, 97-101 **5c** Medical, family, and psycho-social history including relevant genetic information . . . . . . . . . . . . . . . . 114-134 **5d** Relevant past interventions with outcomes . . . . . . . . . . . . . . . . . . . . . . . . . . . . . . . . . . . . . . . . . . . . . . . . . . . . . . . . 89-96, 156-161

**6** Describe significant physical examination (PE) and important clinical findings. . . . . . . . . . . . . . . . . . . . . . . 101-108

**7** Historical and current information from this episode of care organized as a timeline . . . . . . . . . . . . . . . 79-80 **8a** Diagnostic testing (such as PE, laboratory testing, imaging, surveys). . . . . . . . . . . . . . . . . . . . . . . . . . . . . . .87-96, 103-108, 135-150 **8b** Diagnostic challenges (such as access to testing, financial, or cultural) . . . . . . . . . . . . . . . . . . . . . . . . . . . . . 115-125 **8c** Diagnosis (including other diagnoses considered) . . . . . . . . . . . . . . . . . . . . . . . . . . . . . . . . . . . . . . . . . . . . . . . . .91-96, 118-120, 126-134 **8d** Prognosis (such as staging in oncology) where applicable . . . . . . . . . . . . . . . . . . . . . . . . . . . . . . . . . . . . . . . . . 172-177 **9a** Types of therapeutic intervention (such as pharmacologic, surgical, preventive, self-care) . . . . . . . . . . . . . . . . . . . . 153–162 **9b** Administration of therapeutic intervention (such as dosage, strength, duration) . . . . . . . . . . . . . . . . . . . . . . . . . . . . . 154-157, 160-162 **9c** Changes in therapeutic intervention (with rationale) . . . . . . . . . . . . . . . . . . . . . . . . . . . . . . . . . . . . . . . . . . . . . . . . . . . . 158–162

**10a** Clinician and patient-assessed outcomes (if available) . . . . . . . . . . . . . . . . . . . . . . .. . . . . . . . . . . . . . . . . . . . . . . . . . . . 163–171, 306-311 **10b** Important follow-up diagnostic and other test results . . . . . . . . . . . . . . . . . . . . . . . . . . . . . . . . . . . . . . . . . . . . . . . . . . . . 172–175 **10c** Intervention adherence and tolerability (How was this assessed?) . . . . . . . . . . . . . . . . . . . . . . . . . . . . . . . . . . . . . . . . . 166-168 **10d** Adverse and unanticipated events . . . . . . . . . . . . . . . . . . . . . . . . . . . . . . . . . . . . . . . . . . . . . . . . . . . . . . . . . . . . . . . . . . . 89-96, 109-111 **11a** A scientific discussion of the strengths AND limitations associated with this case report . . . . . . . . . . . . . . . . . . . . . . . 292–298 **11b** Discussion of the relevant medical literature **with references**. . . . . . . . . . . . . . . . . . . . . . . . . . . . . . . . . . . . . . . . . . 188–295, 276-258 **11c** The scientific rationale for any conclusions (including assessment of possible causes) . . . . . . . . . . . . . . . . . . . . . . . . 263-274 **11d** The primary “take-away” lessons of this case report (without references) in a one paragraph conclusion . . . . . . . 313–323


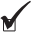
**12** The patient should share their perspective in one to two paragraphs on the treatment(s) they received . . . . . . . . 306–311, 333-339

**13** Did the patient give informed consent? Please provide if requested . . . . . . . . . . . . . . . . . . . . . . . . . . . . . . . . . . . . . . **Yes**
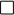
 **No**
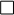

Supplement: Supplementary file 1 — Supplementary Material 1. [file 13223_2026_1011_MOESM1_ESM.docx]
